# Fatigue in patients with inflammatory bowel disease—strongly influenced by depression and not identifiable through laboratory testing: a cross-sectional survey study

**DOI:** 10.1186/s12876-023-02906-0

**Published:** 2023-08-22

**Authors:** Victoria Uhlir, Andreas Stallmach, Philip Christian Grunert

**Affiliations:** Department of Internal Medicine IV, Jena University Hospital, Universitätsklinikum Jena, Am Klinikum 1, 07747 Jena, Germany

**Keywords:** Fatigue, Crohn’s disease, Ulcerative colitis, Depression

## Abstract

**Background:**

Fatigue is a debilitating and highly relevant symptom in patients with inflammatory bowel disease (IBD). However, awareness of fatigue and treatment options remains limited. This study was aimed at elucidating the influence of disease activity and common complications (pain, anemia, depression, anxiety and quality of life) on fatigue in patients with IBD to identify potential interventional targets for treating physicians.

**Methods:**

A cross-sectional survey including five questionnaires (HADS, Fatigue Assessment Scale, McGill Pain Questionnaire, IBDQ and general well-being) was performed on patients with IBD (*n* = 250) at a university IBD clinic. Additionally, demographic data, laboratory data, IBD history, treatment and current disease activity (Harvey-Bradshaw Index, partial Mayo Score, calprotectin and CRP) were recorded.

**Results:**

A total of 189 patients were analyzed (59.8% with Crohn’s disease (CD) and 40.2% with ulcerative colitis (UC)). A total of 51.3% were fatigued, and 12.2% were extremely fatigued. Multiple factors showed significant correlations in univariate analysis. Multivariate analysis revealed that fatigue was correlated with depression (CD, *p* = 0.002; UC, *p* = 0.02), diminished quality of life (CD, *p* = 0.015), female sex (CD, *p* = 0.015) and younger age (UC, *p* = 0.024), whereas the influence of anemia or disease activity was non-significant.

**Conclusions:**

Fatigue is burdensome and highly prevalent in patients with active and inactive IBD. Considerations for fatigue treatment, beyond targeting inflammation and anemia, should include investigation of underlying sub-clinical depression.

**Supplementary Information:**

The online version contains supplementary material available at 10.1186/s12876-023-02906-0.

## Background

Inflammatory bowel diseases (IBDs) are relapsing inflammatory bowel conditions characterized by unclear etiology and an unpredictable disease course. In addition to inflammatory activity, psychosocial symptoms such as fatigue, persistent and extreme forms of mental and/or physical exhaustion, tiredness or weakness occur [1]. In some patients, the psychosocial symptoms are more debilitating than the underlying somatic disease itself [2]. A lack of energy, the cardinal symptom of fatigue, is one of the most frequent symptoms in IBD. As many as 80% of patients with active disease and about 60% of patients in remission report this symptom and find it even more debilitating than urgency, diarrhea, flatulence and pain [3]. If left untreated, fatigue can increase over time [4]. Further studies from the USA and France have reported similar high incidence of fatigue (45–65%) in Crohn’s disease (CD) and ulcerative colitis (UC) [5, 6], at rates comparable to those observed with other chronic diseases such as cancer, multiple sclerosis or rheumatoid arthritis [7].

Although fatigue is highly relevant [3, 8], it is not well understood by patients or practitioners [9], and it has received insufficient attention in previous years. One reason for the low recognition of fatigue in clinical consultations might be that fatigue assessment is challenging and not clearly defined. Particularly in time-limited outpatient visits, the psychosocial aspects of disease may be neglected, despite their high importance in patients with IBD [10].

In previous studies, fatigue has been associated with depression [11–13], pain [14], anxiety [15, 16], low quality of life [12, 17], psychological well-being [4, 18], anemia [8, 19], and sex [20, 21]. The influence of disease activity is controversial. Some studies have reported that disease activity is a relevant factor [16, 22]. Physicians’ current management of fatigue consists primarily of addressing inflammatory activity and nutritional deficits. However, fatigue is highly prevalent even in quiescent disease [2, 7, 23], at levels above those in healthy controls [2], and an increase in fatigue over time is independent of disease pattern [4]. Thus, the causes can be assumed to be multifactorial, and the dependence of fatigue on potential influencing factors must be ruled out.

The aim of this study was to further elucidate the influence of disease activity and common complications, such as pain, anemia, depression, anxiety and decreased quality of life, on fatigue in patients with IBD, to identify potential interventional targets for daily practice. In a cross-sectional study, a sizeable IBD cohort was assessed with the Fatigue Assessment Scale (FAS), IBD disease activity clinical scores, paraclinical activity parameters and a large set of validated questionnaires.

## Patients and methods

### Study population

This cross-sectional paper-based survey study was performed at the Department of Internal Medicine, Jena University Hospital, a third-level IBD clinic with approximately 2,100 patients with IBD. Patients were recruited by the hospital staff during outpatient visits. The inclusion criteria were 1) patients 18 years of age and older, 2) diagnosis of Crohn`s disease (CD) or ulcerative colitis (UC) and 3) German language proficiency.

### Data collection

A total of 250 patients were asked to participate in the study between June 27, 2018 and November 11, 2018. After providing written consent, the patients completed five questionnaires evaluating fatigue, depression and anxiety, pain, health related quality of life and general well-being. Additionally, demographic information on sex, age, occupation and current medications was recorded. Disease activity was assessed by the treating physician. Laboratory values were obtained from blood results measured no more than 4 weeks before or after the appointment. Patients were included in the analysis when at least 95% of questionnaire items were completed.

### Measurements and instruments

#### Clinical evaluation of disease activity

Disease activity in CD was assessed with the Harvey-Bradshaw Index (HBI) [24]. Scores < 5 points indicated remission, 5–7 points indicated mild disease, and higher scores indicated severe disease. UC disease activity was graded with the partial Mayo Score (pMayo) [25]. Scores < 2 points indicated remission, 2–4 points indicated mild disease, 5–6 points indicated moderate disease, and 7–9 points indicated severe disease. Because the UC population was relatively small, we decided to include patients with moderate to severe disease in one group, to achieve better evaluation quality.

#### Ascertainment of fatigue

The FAS is a unidimensional ten item fatigue scale, which has previously been used for the general public [26, 27] and for more than 26 other chronic diseases [27–30]. Each item is rated from 1 (never) to 5 (always), thus resulting in a total score ranging from 10 to 50 points, with 10–21 points indicating no fatigue, 22–34 points indicating fatigue and ≥ 35 points indicating extreme fatigue. In a comparison of six different fatigue questionnaires, the FAS has been found to be the most promising measure of fatigue with good reliability and validity [26].

#### Ascertainment of anxiety and depression

Anxiety and depression were assessed with the German version of the Hospital Anxiety and Depression Scale, a 14-item self-reported questionnaire with seven items each for the dimensions of depression (HADS-D) and anxiety (HADS-A). Each item is rated 0–3 according to severity, thus resulting in scores of 0–21 for each dimension [15]. A score of 8–10 indicates suspicion, and a score > 10 points indicates a disorder, of either anxiety or depression. This scale has been widely used in patients with IBD [11, 13, 15, 16].

#### Ascertainment of quality of life

The Inflammatory Bowel Disease Questionnaire (IBDQ) is a disease specific instrument used to assess the health-associated quality of life (QoL) in patients with IBD. We used the German version, which has been validated by Janke et al. It contains 32 items, each ranging from 1 (worst) to 7 (best), and assesses the QoL in four dimensions (bowel, systemic, emotional and social well-being) and provides a total score. Higher scores indicate better QoL [31]. For our correlations, we used only the total score.

#### Ascertainment of pain

The Short-Form McGill Pain Questionnaire is a well-established and widely used measure to evaluate pain [32, 33]. It consists of three parts. The first part assesses pain with adjectives, each graded from 0 (none) to 3 (severe), thus illustrating the sensory (11 Items) and affective (4 Items) component of pain (pain rating index). In the second part, pain is quantified on a 0–100 Visual analogue Scale (VAS). The present pain intensity is graded on a 5-point scale from 0 (no pain) to 5 (excruciating) in the third part. The scores for the questionnaire can range from 0 to 45 on the pain rating index; from 0 to 10 cm on the VAS; and from 0 to 5 on present pain intensity.

#### Ascertainment of general well-being

Patients were asked to rate their current general well-being on a Global Assessment Scale ranging from 0 (very poor) to 100 (very good).

#### Clinical laboratory values

To identify anemia, we evaluated hemoglobin (Hb), mean corpuscular volume and mean corpuscular hemoglobin. Systemic inflammation was assessed on the basis of the C-reactive protein (CRP), which we rated as normal for all values under 7 mg/l. For IBD specific inflammation, we used fecal calprotectin, a surrogate parameter for mucosal inflammation. Values under 100 mg/kg stool were rated as normal/remission, whereas values above 100 mg/kg indicated mucosal inflammation [34].

### Data analysis

Descriptive analysis was performed with simple statistical standards, such as percentages and mean values. Bar charts, histograms, and box plots with percentages were used for presentation.

All metric scaled scores and age were tested with the determined fatigue score with Pearson`s correlation coefficient and are presented in scatter plots. Values above 0.5 indicated a strong effect.

For mean value comparison, the variables to be tested were divided into groups, which were tested against each other on the basis of the achieved fatigue score. The groupings were as follows.

Laboratory values for CRP and fecal calprotectin: "normal" vs "elevated"; anemia: "yes" vs "no"; sex: "male" vs "female"; diagnosis: "CD" vs "UC"; disease activity: "remission" vs "moderate to severe disease"; anxiety and depression: "inconspicuous" vs "conspicuous, severe to very severe symptoms"; and overall assessment of intensity of pain: "non-low/mild" vs “distressing or worse.” Group analysis was performed with the Mann–Whitney U-test. *P* values < 0.05 were considered significant. The Kruskal–Wallis test was used to assess fatigue differences within the disease location, behaviour and age at diagnosis groups.

For nominal (anemia, CRP elevation or fecal calprotectin elevation) or ordinal (anxiety, depression, disease activity, quality of life or total pain) variables, rank chi squared test (with trend test from three variables) was performed, and cross tables were created. *P* values < 0.05 were considered significant.

To avoid a high number of missing cases in multiple linear regression analysis, we conducted a univariate analysis for each variable to screen for significant factors correlating with fatigue. For all metric scores (HBI, pMayo, HADS-A/D, IBDQ, current pain, total pain and GAS) and age, Pearson´s correlation coefficient was used. All nominal factors (anemia, CRP elevation, fecal calprotectin elevation, sex and diagnosis, surgery, medication, stoma) were tested by median comparison with Mann–Whitney U-test. All variables showing a significant correlation (*p* < 0.05) in the univariate analysis, as well as sex and age, were included in multiple linear regression analysis. Multiple regression analysis was performed for CD and UC separately. All variables were tested for collinearity. Analyses were performed in IBM SPSS Statistics version 25 (IBM Corp., Armonk, NY, USA).

## Ethical considerations

Ethical approval was granted by the Ethics Committee of the Jena University Hospital, Jena, Germany (5530–05/18), and all patients provided signed informed consent, according to the Declaration of Helsinki.

## Results

A total of 250 patients were asked to participate in the study; 210 patients consented to participate, among whom a total of 189 patients (76% response rate) completed at least 95% of the questionnaire items and thus were included in the analysis. A total of 113 (59.8%) patients had CD, and 76 (40.2%) patients had UC. Clinical and socio-demographic characteristics are presented in Table 1. The results of psychometric testing and laboratory values classified by type of IBD are shown in Table 2.

**Table 1:** Socio-demographic characteristics

	**CD**	**N**^**a**^	**UC**	**N**^**a**^	**Total**	**N**^**a**^
**Socio-demographic characteristics**						
Total number of patients	113 (59.8%)		76 (40.2%)		**189**	189
Age	38 (18 -74)		47 (19–80)		**40 (18–80)**	189
Sex female/male	59/54		30/46		**89/100**	189
Employed	73 (67.6%)	108	45 (62.5%)	72	**118 (65.6%)**	180
**Disease activity (HBI/partial Mayo)**		104		71		175
Remission	59 (56.7%)		32 (45.1%)		**91 (52.0%)**	
Mild disease	22 (21.2%)		23 (32.4%)		**45 (25.7%)**	
Moderate to severe disease	23 (22.1%)		16 (22.5%)		**39 (22.3%)**	
**IBD Characteristics (Montreal)**		112		76		
**Age at Diagnosis**						
A1 < 17 years	21 (18.8%)					
A2 17–40 years	68 (60.7%)					
A3 > 40 years	23 (20.5%)					
**Location (CD = L, UC = E)**						
L1 Terminal Ileum / E1 Proctitis	22 (19.6%)		2 (2.6%)			
L2 Colon / E2 Left-sided	28 (25%)		41 (53.9%)			
L3 Ileocolonic / E3 Pancolitis	47 (42%)		29 (38.2%)			
Upper and lower GI-tract (+ L4) / Ileal pouch	15 (13.4%)		4 (5.3%)			
**Behavior**						
B1 non-stricturing/non-penetrating	39 (34.8%)					
B2 stricturing	30 (26.8%)					
B3 penetrating	16 (14.3%)					
B3p perianal disease	27 (24.1%)					

^a^*N* number of patients included for analysis

**Table 2:** Results of psychometric tests and laboratory values

	**CD**	**SD**	**Min**	**Max**	**UC**	**SD**	**Min**	**Max**	**Total**	**SD**	**Min**	**Max**
**Psychosocial factors (N**^**a**^**)**												
FAS (189)												
No fatigue	41 (36.3%)				28 (36.8%)				**69 (36.5%)**			
Fatigue	60 (53.1%)				37 (48.7%)				**97 (51.3%)**			
Extreme fatigue	12 (10.6%)				11 (14.5%)				**23 (12.2%)**			
Mean value fatigue score	24.72	7.27	10	40	24.71	7.54	12	43	**24.71**	7.36	10	43
IBDQ < 171 (active disease) (186)	66 (58.4%)				43 (56.6%)				**109 (58.6%)**			
IBDQ score in points^b^	158	35	87	222	157.1	36.5	69	213	**157.6**	35.5	69	222
GAS 0–100 in points^b^ (178)	68.3	22.6	10	100	66.9	23.9	10	99	**67.7**	23.1	10	100
HADS-A (anxiety) ^b^ (187)	6.68	4.1	0	16	6.96	4.4	0	18	**6.79**	4.2	0	18
Indicating anxiety (> 10)	20 (17.9%)				18 (24.0%)				**38 (20.3%)**			
HADS-D (depression)^b^ (187)	5.04	4.1	0	17	5.41	3.78	0	16	**5.19**	3.96	0	17
Indicating depression (> 10)	15 (13.5%)				7 (9.2%)				**22 (11.8%)**			
McGill Pain Questionnaire (179)												
Total pain none/ low – mild	35 (32.7%)				26 (36.1%)				**61 (34.1%)**			
Total pain disstressing or worse	50 (46.7%)				32 (44.5%)				**82 (45.8%)**			
Sensoric component of pain^b^ (181)	5.59	5.4	0	21	4.75	5.78	0	25	**5.25**	5.56	0	25
Affective component of pain^b^ (181)	2.63	3.08	0	12	2.12	2.56	0	11	**2.43**	2.89	0	12
Current pain on VAS^b^ (182)	1.94	2.26	0	8.3	1.16	1.73	0	7.2	**1.6**	2.06	0	8.3
**Laboratory values (N**^**a**^)												
Anaemia (169)	33 (33.0%)				28 (40.6%)				**61 (36.1%)**			
CRP > 7 (163)	28 (28.9%)				16 (24.2%)				**44 (27.0%)**			
Fecal calprotectin > 100 mg/kg (60)	18 (46.2%)				17 (81.0%)				**35 (58.3%)**			

^a^*N* = number of patients included for analysis

^b^ Mean value

### Prevalence and severity of fatigue

**Fig. 1:**
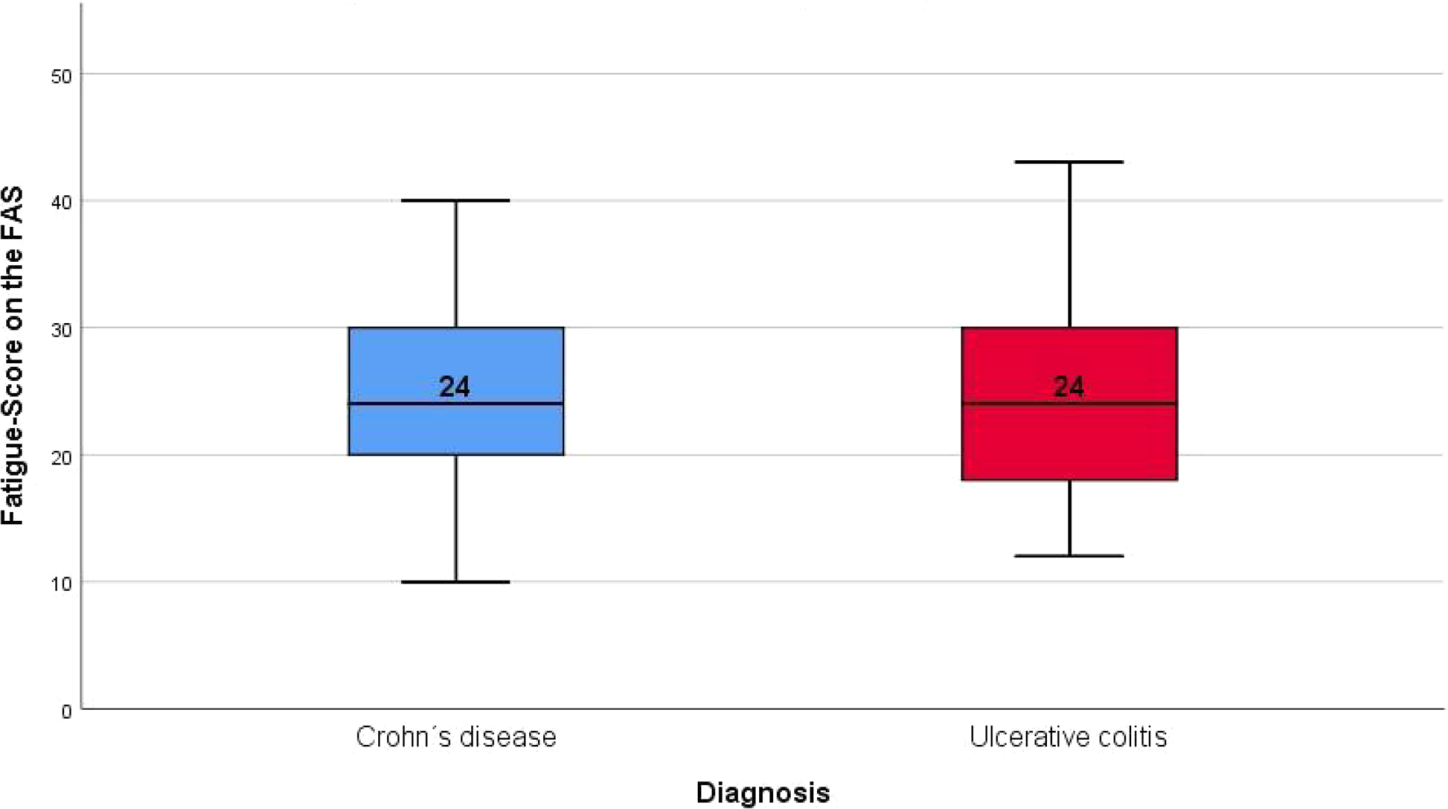
Mean fatigue scores (FAS), separated by IBD type. Data is displayed as grouped boxplots. *N* = 189

**Fig. 2:**
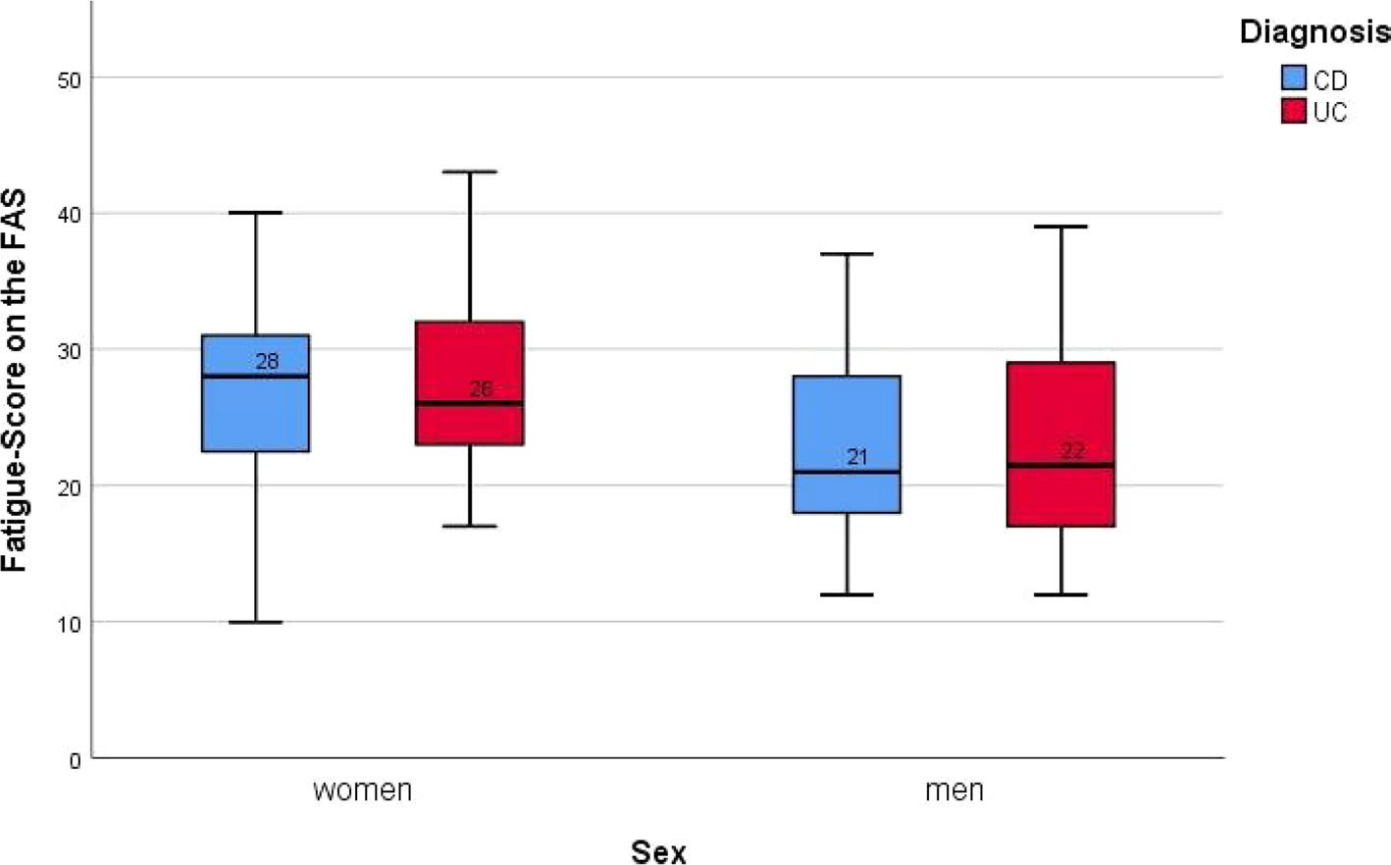
Mean fatigue scores (FAS), separated by IBD type and sex. Data is displayed as grouped boxplots. *N* = 189

The mean FAS fatigue scores were 24.72 (median: 24, IQR: 19,5–30) and 24.71 (median 24, IQR: 18–30,5) for CD and UC, respectively (Fig. 1), and no statistical difference between diagnoses was observed (*p* = 0.838, Table 3). Two-thirds of patients were either fatigued (CD: 53.1%, UC: 48.7%) or extremely fatigued (CD: 10.7%, UC: 14.5%). Fatigue was significantly more frequent in women (80,9%) than in men (48,0%) (median fatigue score 27 vs 21, *p* < 0.001, Table 3, Fig. 2). Because we did not find significant differences across IBD subtypes in univariate analyses for all factors, we decided not to distinguish by diagnosis, except for disease activity, for clarity of presentation in the tables.

**Table 3:** Total fatigue score, tested against demographic factors/laboratory values with Mann–Whitney U-test

		**Total fatigue score value**	***p*-value**
**Factors**	**N**^**a**^	**median (IQR)**	
Anemia			
No	108	24 (19–30.75)	0.538
Yes	61	24 (20–29.5)	
CRP			
Normal	119	24 (19–31)	0.109
Elevated	44	22.5 (18–28)	
Fecal calprotectin			
Normal	25	25 (20–30.5)	0.898
Elevated	35	24 (19–31)	
Sex			
Female	89	27 (23–31.5)	**< 0.001**
Male	100	21 (18–28)	
Diagnosis			
CD	113	24 (19.5–30)	0.838
UC	76	24 (18–30.5)	
Employment status			
Employed^b^	134	24 (20–29)	0.357
Unemployed^c^	46	25.5 (18–33)	

*p*-value = highly significant *p* < 0.001

N^a^ = number of patients included for analysis

Employed^b^ = employed, students and trainees

Unemployed^c^ = unemployed and retired

### Fatigue and disease characteristics

Fatigue was more often present in patients with moderate to severe disease activity than in patients in remission (CD: 95.7% vs 46.6%, UC: 75% vs 62.5%). This finding was significant in only CD (*p* < 0.001) but not UC (*p* = 0.80). In CD, 82.1% of the patients without fatigue were in remission, whereas 54.5% of those with extreme fatigue had moderate to severe disease. This weak positive correlation was statistically significant in univariate analysis (*p* < 0.001). Regarding disease characteristics beyond disease activity, no correlations were found between fatigue and age at diagnosis (H(2) = 0.183, *p* = 0.913), disease location (H(3) = 1.09, *p* = 0.779), or disease behaviour (H(3) = 2.36, *p* = 0.501) in patients with Crohn's disease (CD) or disease location (H(3) = 0.507, *p* = 0.917) in patients with ulcerative colitis (UC). In the HBI documented complications for CD (fistulas, erythema nodosum etc.) did not correlate with higher levels of fatigue. 22 patients reported having undergone bowel surgery. There was no significant influence of surgery on fatigue (*p* = 0.864). 5 patients had a stoma which also did not impact fatigue scores (*p* = 0.881). IBD specific medication did not influence fatigue levels (any IBD medication *n* = 162, *p* = 0.108; biologics *n* = 89, *p* = 0.351; mesalazine *n* = 64, *p* = 0.555; thiopurines *n* = 28, *p* = 0.439; steroids *n* = 48, *p* = 0.724).

### Psychosocial factors associated with fatigue

**Fig. 3:**
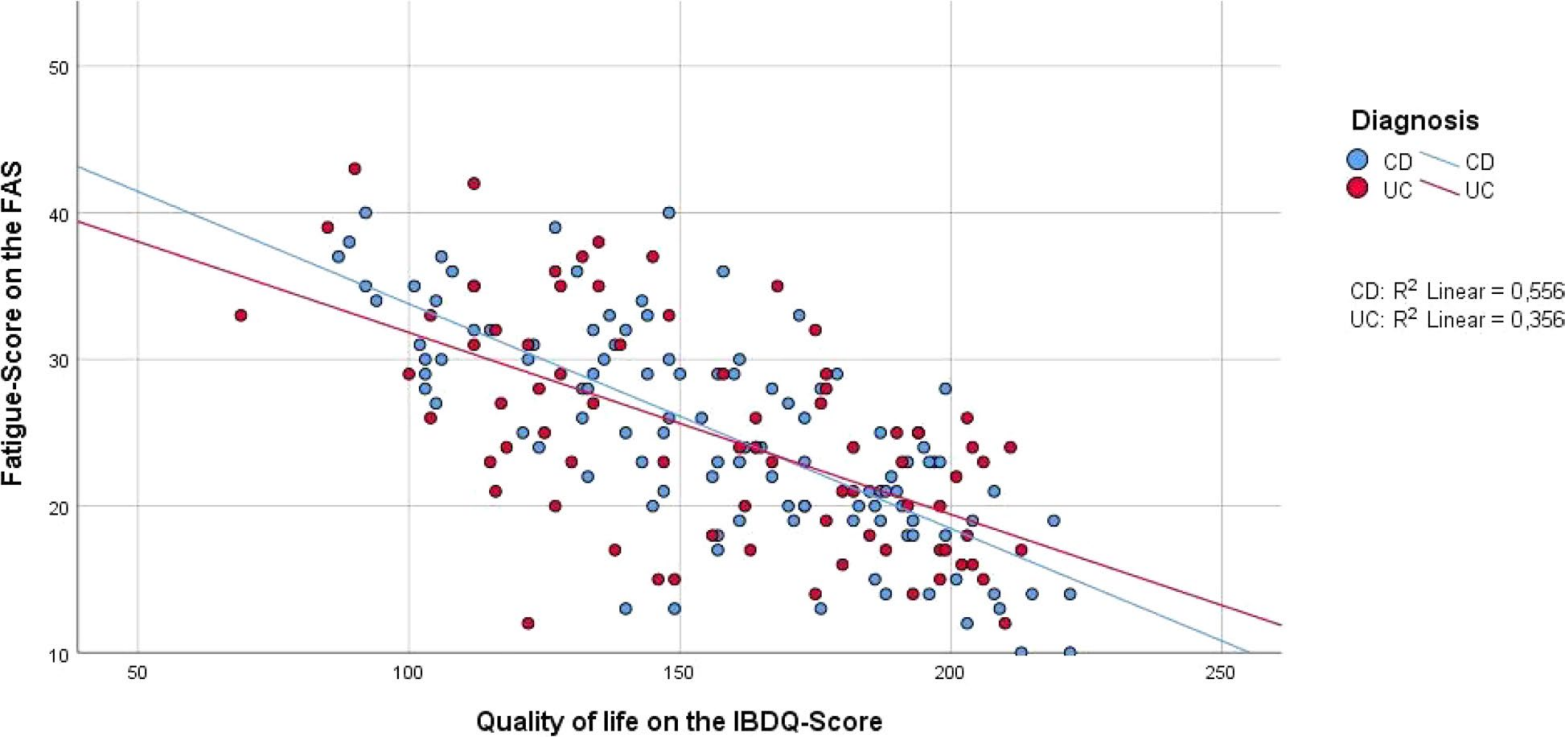
Correlation of fatigue score (FAS) and quality of life score (IBDQ), separated by diagnosis. Data is displayed as grouped scatter plot. *N* = 186

**Fig. 4:**
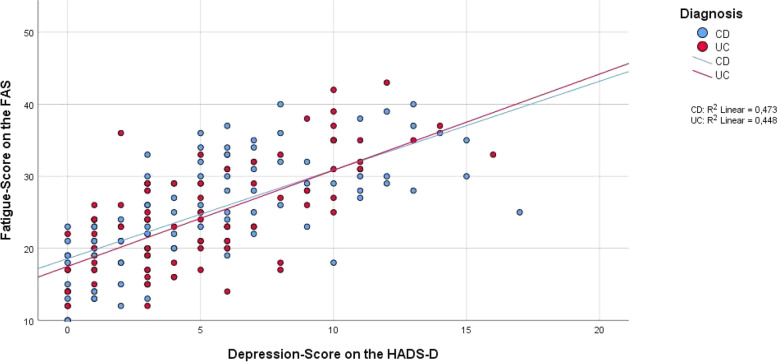
Correlation of fatigue score (FAS) and depression score (HADS-D), separated by diagnosis. Data is displayed as grouped scatter plot. *N* = 187

Univariate factor analysis with Pearson´s correlation coefficient showed that anxiety, depression, health related quality of life, general well-being and pain were associated with the presence of fatigue and total fatigue score. The strongest correlations were found for quality of life and depression (Table 4, Figs. 3 and 4).

**Table 4:** Correlations among total scores of disease activity, psychosocial factors and age, and the FAS score

		**Total fatigue score value**		
**Total score value**	**N**^**a**^	**Pearson correlation**	***p*-value**	**R**^**2**^**- value**
HBI (for CD)	104	0.466	**< 0.001**	0.217
Partial Mayo Score (for UC)	71	0.183	0.183	0.026
HADS-A	187	0.616	**< 0.001**	0.379
HADS-D	187	0.679	**< 0.001**	0.46
IBDQ	186	-0.682	**< 0.001**	0.465
Current pain (McGill-Pain-Q.)	182	0.358	**< 0.001**	0.128
Total pain (McGill-Pain-Q.)	181	0.39	**< 0.001**	0.152
GAS	178	-0.562	**< 0.001**	0.316
Age	189	-0.012	0.869	1.449

*p*-value = highly significant *p* < 0.001

^a^N = number of patients included for analysis

### Fatigue and anxiety

Patients without conspicuous anxiety symptoms had significantly lower mean fatigue scores than patients with conspicuous anxiety symptoms (median fatigue scores: 21 vs 32, *p* < 0.001). A total of 83.8% of patients without fatigue showed unremarkable anxiety symptoms, whereas 60.9% of patients with extreme fatigue were classified as anxious with HADS-A (*p* < 0.001).

### Fatigue and depression

Similar strong effects were found regarding depression. Whereas patients without depression had median fatigue scores of 22 points, the group classified as depressed according to HADS-D had a median fatigue score of 33 points (*p* < 0.001). Whereas 95.6% of the group without fatigue had no quantifiable depressive symptoms, the depression was severe to very severe in 43.5% of the extremely fatigued group.

### Fatigue and HRQoL

Patients with fatigue or extreme fatigue significantly more often had low scores for the magnitude of active disease (IBDQ < 171) (extreme fatigue: 100%, fatigue: 71.9%, no fatigue: 25.4%, *p* < 0.001). A total of 64.9% of the patients with IBDQ scores > 171 (suggestive of remission) had “no fatigue.” In contrast, 84.4% of the patients with low IBDQ scores experienced either fatigue or extreme fatigue. The majority of the 32 items and all of the 4 domains of the IBDQ showed a significant correlation with fatigue, as determined by Pearson’s correlation coefficient (Additional file 1). There were some exceptions, including UC items 1 (stool frequency), 5 (loose stools), 7 (worried about surgery), 17 (passing gas), 22 (rectal bleeding) as well as 26 (soiling), and CD item 22 (rectal bleeding). The strongest correlations for fatigue were observed for item 2 (tired / worn out) (UC: r(74) = -0.717, *p* =  < 0.001; CD: r(111) = -0.750, *p* =  < 0.001), item 6 (energy) (UC: r(74) = -0.655, *p* =  < 0.001); CD: r(110) = -0.641, *p* =  < 0.001) and item 15 (depressed / discouraged) (UC: r(74) = -0.686, *p* =  < 0.001;CD: r(112) = -0.653, *p* =  < 0.001).

### Fatigue and general well-being

Although a wider variation was observed than that in other factors, a strongly significant, anti-proportional correlation was found (R^2^ = 0,316, *p* < 0.001, Table 4) between the total fatigue score and the GAS score.

### Fatigue and pain

Pearson's correlation coefficient indicated linear correlations of current pain intensity on the VAS (R^2^ = 0.128, *p* < 0.001, Table 4) with the total verbal pain score (R^2^ = 0.152, *p* < 0.001, Table 4) and the total fatigue score. The subjective overall assessment of the pain intensity caused by the disease indicated that 52.3% of patients who did not have fatigue classified their pain as low or mild. In contrast, 77.2% of the patients with extreme fatigue reported that their pain intensity was distressing or worse (*p* < 0.001).

### Fatigue and laboratory values

Univariate factor analysis of the total fatigue score and laboratory values is shown in Table 3. No statistical differences were found regarding anemia, elevated CRP or elevated fecal calprotectin. A total of 36.1% (61) of participants had anemia. Analysis according to fatigue indicated no significant difference between groups: 62.3% of the anemic patients and 63.9% of the non-anemic patients had fatigue (*p* = 0.437). In addition, the inflammatory values in the blood and stool were not found to be predictive factors for fatigue (*p* = 0.057; *p* = 0.27).

### Multivariate analysis

In the multivariate analysis, we stratified by IBD diagnosis and included all factors that were significant in the univariate analysis as well as sex and age (Table 5). After controlling for each of the other variables, sex (*p* = 0.015), depression (*p* = 0.002) and quality of life (*p* = 0.003) in CD, and age (*p* = 0.024) and depression (0.02) in UC were significantly associated with fatigue. Trends were observed for age (*p* = 0.057) in CD and for anxiety (*p* = 0.055) in UC. Unexpectedly, an antiproportional trend was found for disease activity and fatigue in UC. All other factors were found to be confounders. The results are shown in Table 5. Although association with sex was highly significant over both diagnoses in univariate analysis, no correlation was observed for UC in regression analysis. In contrast, age was not significant in the univariate analysis but was significant in regression analysis for CD.

**Table 5:** Multiple linear regression analysis of factors associated with total fatigue score

	Crohn´s disease		Ulcerative colitis	
Factors	Regression coefficient B (95%CI)	*p*-value*	Regression coefficient B (95%CI)	*p*-value*
Female vs male	2.333 (0.466–4.199)	0.015	1.603 (-1.270–4.476)	0.268
Age	0.067 (0.002–0.136)	0.057	-0.109 (-0.202—0.015)	0.024
HBI/Partial Mayo Score	-0.223 (-0.535–0.090)	0.160	-0.593 (-1.270–0.084)	0.085
HADS-D	0.613 (0.231–0.996)	0.002	0.756 (0.126–1.386)	0.020
HADS-A	0.177 (-0.187–0.541)	0.337	0.470 (-0.011–0.950)	0.055
IBDQ	-0.113 (-0.186–-0.041)	0.003	-0.032 (-0.110–0.046)	0.415
GAS	0.010 (-0.063–0.082)	0.794	-0.019 (-0.105–0.067)	0.663
Total pain (McGill-Pain-Q.)	0.058 (-0.104–0.221)	0.477	-0.051 (-0.246–0.145)	0.606
Current pain (McGill-Pain-Q.)	-0.168 (-0.949–0.613)	0.670	0.576 (-0.399–1.546)	0.239

^*^ = significant for all *p* < 0.05

## Discussion

Fatigue in patients with IBD remains poorly understood. This cross-sectional study of 189 patients with IBD, aimed to investigate the prevalence of fatigue, explore its associations with various factors, and identify important implications for clinical practice. It indicated three major findings. First, fatigue was highly prevalent: 63.5% of patients with IBD experienced either fatigue or severe fatigue, regardless of disease type and activity. Second, to address fatigue, physicians should look beyond laboratory parameters, because anemia and lab values which reflect disease activity do not reliably correlate with fatigue. Third, the diagnostic workup of patients presenting with fatigue should include screening for depression.

In this study, the observed prevalence of fatigue in patients with IBD was 63.5% (51.3% with fatigue and 12.3% with severe fatigue)—a percentage four times higher than that reported in the general population [35]. The FAS results in patients with IBD are consistent with those in previously published studies using other fatigue instruments [5, 6, 19, 36]. In line with findings from previous studies, no differences between the type of IBD and the occurrence or severity of fatigue were found [15, 16, 19, 22]. The prevalence of fatigue in patients with IBD in remission was approximately 50% in our study. Nearly all past studies have reported that a large number of patients in remission still have fatigue [4, 13, 15]. Therefore, the fatigue response may be independent of the degree of intestinal inflammation and may be signaled through other pathways [13].

Management of fatigue in patients with IBD is challenging. If following guidelines, laboratory testing for, and supplementing with, vitamin B12, folate and iron are recommended [37].

Anemia is one of the most frequent complications in patients with IBD [38, 39]. However, in this study, no association was found between the presence of anemia and fatigue, as 62.3% of the anemic patients and 63.9% of the non-anemic patients experienced fatigue. Conflicting results have been reported [2, 8, 9, 37, 40], but, the majority of studies are in agreement with our findings [13, 15, 19, 22, 41–43]. Vitamin B12, folate and iron levels were not assessed in the current study, thus, subclinical deficiencies may have an impact. Likewise, a recent randomized controlled trial demonstrated the effectiveness of high-dose oral thiamine for the treatment of fatigue in patients with quiescent IBD [44].

The influence of disease activity on fatigue development has been investigated in previous studies. Most studies that performed multivariate regression analysis, to account for possible confounders, did not find an association between fatigue and disease activity, in accordance with our data [2, 13, 15, 19, 45, 46]. Among the studies that found associations for IBD [4, 17, 42, 47] or specific subtypes of IBD [16, 48], two studies did not correct for psychosomatic dimensions (e.g., pain, anxiety and depression) [17, 42], and those that did correct have found conflicting results [4] or no correlation with disease activity after multivariate analysis [47, 48]. Studies that have used the same activity scores (HBI and partial Mayo) have come to the same conclusion that disease activity alone is not the main driver of fatigue [13, 15].

In agreement with the observations of clinical scores, fecal calprotectin, which correlates well with disease activity [34], and CRP elevation did not show a correlation with fatigue in both CD or UC in this study. This finding is consistent with previous studies [16, 22, 42, 47]. Huppertz-Hauss et al. and Bager et al. have described that the perceived disease’s symptom burden, rather than its biochemical markers, correlates with fatigue [16, 42]. Further analysis of the IBDQ results in this study revealed that patient-reported items indicating active disease, such as rectal bleeding (UC/CD), loose stools (UC), and increased stool frequency (UC) did not show a significant correlation with the level of fatigue. However, items related to disease burden, such as disrupted / restricted participation in activities, embarrassment and emotional aspects did correlate significantly. The findings of Jelsness-Jorgensen et al. indicating that patients with inactive IBD and additional irritable bowel syndrome have higher fatigue levels than those without, underlines the influence of perceived burden rather than actual inflammatory activity [49].

In our study, depression, measured by the HADS-D, showed the strongest associations with fatigue in both CD and UC in the multivariate analysis. Similar results have been reported by previous studies [7, 13, 15, 46, 50, 51]. Depression and fatigue have common behavioral, affective and cognitive characteristics; furthermore, they may even share similar causal pathways [7, 46, 51]. Truyens et al. [52] suspected fatigue to be a symptom of underlying undetected depression. This hypothesis was supported by our study, in which 100% of patients with conspicuous values on the HADS-D scale had fatigue. The connection between depression and IBD through the gut-brain-axis has been shown to be bidirectional. Severe IBD has been shown to impact psychology and is associated with new-onset depression [53]. Conversely, depression can negatively affect the course of IBD [52, 54–56]. Hence identifying and appropriately addressing depression in patients with IBD are crucial. Patients reporting symptoms of fatigue should be screened for concomitant depression, particularly when the common fatigue work-up is unremarkable.

Despite strong correlations in the univariate analysis, the variables anxiety, current well-being and pain did not remain significant in the multivariate analysis and were found to be confounders. Nevertheless, they should not be completely disregarded in the assessment of fatigue, regarding anxiety there was a tendency for correlation in the multivariate analysis in UC. Age-related effects on fatigue exhibited a weak and inconsistent correlation, possibly stemming from variations in age and gender distribution between the two disease types [42].

As in any scientific investigation, this study has several limitations. The participants in this study were patients from the outpatient department of a tertiary referral center. This may limit the generalizability of the findings to the entire IBD population, e.g., the level of fatigue among outpatients in an oncological study was observed to be higher compared to that of inpatients [57]. Laboratory values were recorded not numerically but only nominally (increased or not increased). In addition, the data were laboratory values from a maximum of 4 weeks before or after completion of the questionnaires, thus potentially leading to inaccuracy, given that therapeutic interventions or flares might have occurred during the period between sample collection and assessment of fatigue. Moreover, most surveyed patients were in remission (52%) or had only mild symptoms (25.7% mild disease).

The FAS, which has been shown to be a reliable instrument to assess fatigue [26, 27], is unidimensional, thereby hindering direct comparison with previous IBD studies that have used other (multidimensional) fatigue scores.

The strengths of the study include the broad assessment of possible factors influencing fatigue through a broad set of validated questionnaires as well as clinical and paraclinical data in almost 200 patients with IBD.

## Conclusions

In conclusion, we demonstrated that fatigue is burdensome and highly prevalent in patients with IBD. Fatigue should receive attention in patients with active disease as well as disease in remission, because it did not significantly correlate with anemia or disease activity. Thus, we recommend implementing an easy to fill-out fatigue survey, such as the FAS which takes about 2 min to complete, in daily practice. The strong associations with depression highlight the importance of investigating and addressing possible underlying sub-clinical depression in patients reporting fatigue symptoms.

### Supplementary Information

**Additional file 1.** Correlation between IBDQ items / domains and the total fatigue score by Pearson´s correlation coefficient.

## Data Availability

The datasets used and analysed during the current study are available from the corresponding author on reasonable request.

## Abbreviations

CD: Crohn’s disease
CRP: C-reactive protein
FAS: Fatigue Assessment Scale
GAS: Global Assessment Scale
HADS: Hospital Anxiety and Depression Scale
Hb: Hemoglobin
HBI: Harvey-Bradshaw Index
HRQoL: Health related quality of life
IBD: Inflammatory bowel disease
IBDQ: Inflammatory Bowel Disease Questionnaire
pMayo: Partial Mayo Score
UC: Ulcerative colitis
VAS: Visual analogue scale
