# Supplementary material for: Fatigue in patients with inflammatory bowel disease—strongly influenced by depression and not identifiable through laboratory testing: a cross-sectional survey study
Source: BMC Gastroenterol. 2023 Aug 22;23:288. doi: 10.1186/s12876-023-02906-0 (PMC10463723; doi:10.1186/s12876-023-02906-0)
Supplement: Supplementary file 1 — Additional file 1. Correlation between IBDQ items / domains and the total fatigue score by Pearson´s correlation coefficient. [file 12876_2023_2906_MOESM1_ESM.pdf]

Additional File 1: Correlation between IBDQ items / domains and the total fatigue score by Pearson´s correlation coefficient

	Total Fatigue Score Value							
	Crohn´s disease				Ulcerative colitis			
**IBDQ item / domain**	**N**	**Pearson Correlation**	**p-value***	**R^2-value**	**N**	**Pearson Correlation**	**p-value***	**R^2-value**
Item 1 stool frequency	112	-0.356	**0.001**	0.127	76	-0.203	0.078	0.041
Item 2 tired / worn out	113	-0.750	**0.001**	0.563	76	-0.717	**0.001**	0.514
Item 3 frustrated / impatient	113	-0.603	**0.001**	0.364	76	-0.581	**0.001**	0.338
Item 4 work / school	113	-0.404	**0.001**	0.163	76	-0.455	**0.001**	0.207
Item 5 loose stools	113	-0.396	**0.001**	0.157	76	-0.140	0.229	0.020
Item 6 energy	112	-0.641	**0.001**	0.411	76	-0.655	**0.001**	0.429
Item 7 worried about surgery	113	-0.218	**0.020**	0.046	76	-0,223	0.053	0.050
Item 8 cancel social engagement	113	-0.435	**0.001**	0.189	76	-0.455	**0.001**	0.207
Item 9 abdominal cramps	112	-0.522	**0.001**	0.272	76	-0.354	**0.002**	0.125
Item 10 generally unwell	112	-0.613	**0.001**	0.376	75	-0.561	**0.001**	0.315
Item 11 fear of not finding a toilet	112	-0.508	**0.001**	0.258	75	-0.290	**0.012**	0.084
Item 12 leisure or sports activities	112	-0.612	**0.001**	0.375	75	-0.542	**0.001**	0.294
Item 13 abdominal pain	113	-0.585	**0.001**	0.342	76	-0.455	**0.001**	0.207
Item 14 sleeping disturbance	113	-0.477	**0.001**	0.228	76	-0.357	**0.002**	0.127
Item 15 depressed/discouraged	113	-0.653	**0.001**	0.426	76	-0.686	**0.001**	0.471
Item 16 avoid events without toilet	113	-0.440	**0.001**	0.194	76	-0.397	**0.001**	0.158
Item 17 passing gas	113	-0.445	**0.001**	0.198	76	-0.127	0.275	0.016
Item 18 weight problems	113	-0.313	**0.001**	0.098	76	-0.245	**0.033**	0.060
Item 19 disease-related worries	113	-0.434	**0.001**	0.188	76	-0.367	**0.001**	0.135
Item 20 abdominal bloating	113	-0.549	**0.001**	0.301	76	-0.239	**0.038**	0.057
Item 21 relaxed/free of tension	113	-0.620	**0.001**	0.384	76	-0.465	**0.001**	0.216
Item 22 rectal bleeding	113	-0.176	0.063	0.031	76	-0.214	0.064	0.046
Item 23 embarrassed	113	-0.476	**0.001**	0.227	76	-0.425	**0.001**	0.181
Item 24 feeling of defecation need	113	-0.508	**0.001**	0.258	76	-0.331	**0.003**	0.110
Item 25 tearful or upset	113	-0.619	**0.001**	0.383	76	-0.607	**0.001**	0.368
Item 26 soiling	113	-0.458	**0.001**	0.210	76	-0.201	0.082	0.040
Item 27 angry	113	-0.489	**0.001**	0.240	76	-0.317	**0.005**	0.100
Item 28 sexual activity	113	-0.439	**0.001**	0.193	76	-0.382	**0.001**	0.146
Item 29 sick to your stomach	113	-0.438	**0.001**	0.192	76	-0.322	**0.005**	0.104
Item 30 irritable	113	-0.490	**0.001**	0.240	76	-0.550	**0.001**	0.303
Item 31 lack of understanding	113	-0.454	**0.001**	0.206	76	-0.494	**0.001**	0.244
Item 32 satisfaction with personal life	112	-0.628	**0.001**	0.394	76	-0.531	**0.001**	0.282
Bowel symptoms domain	111	-0.652	**0.001**	0.425	76	-0.372	**0.001**	0.138
Systemic symptoms domain	111	-0.726	**0.001**	0.527	75	-0.682	**0.001**	0.465
Emotional function domain	111	-0.709	**0.001**	0.503	75	-0.603	**0.001**	0.364
Social function domain	112	-0.595	**0.001**	0.354	76	-0.563	**0.001**	0.317
IBDQ total score	111	-0.745	**0.001**	0.555	76	-0.597	**0.001**	0.356

*= significant for all p<0.05
